# Supplementary material for: Fisetin as an Antiviral Agent Targeting the RNA-Dependent RNA Polymerase of SARS-CoV-2: Computational Prediction and In Vitro Experimental Validation
Source: Microorganisms. 2025 Dec 10;13(12):2809. doi: 10.3390/microorganisms13122809 (PMC12736355; doi:10.3390/microorganisms13122809)
Supplement: Supplementary file 1 [file microorganisms-13-02809-s001.zip › microorganisms-3948191-supplementary.pdf]

Supplementary Table S1. Results of the screening and characteristics of the drugs against RNA-Polymerase of SARS-CoV-2. Ligand-target binding energy, toxicity, and drug legislation.

	PubChem CID	Name	Binding Energy (kcal/mol^{-1})		Toxicity (PubChem)	FDA Approved
			Active site RdRp	NiRAN		
NSP12 RNA-polymerase	121304016	Remdesivir (Reference)	-6.81	-7.67	Not available	Yes
	11691726	Balapiravir	-7.25	-8.06	Not available	Yes
	5281614	Fisetin	-7.08	-8.1	Not described in human	Yes
	5281643	Hyperoside	-6.68	-7.43	Not available	Yes

Supplementary Table S2. Non-covalent interactions of drugs and their targets. These were obtained by using the PLIP (Protein-Ligand Interaction Profiler) software.

	Drug	Number of interactions	Hydrogen Bonds	Hydrophobic Interactions	Π-Π Interaction	Salt Bridges
**NSP12 RNA Polymerase (Active site RdRp)**	Remdesivir (Reference)	11	ILE494, ASN496 (2), ASN497, ARG569, GLN573, LEU576, LYS577, TYR689	ASN496		LYS577
	Balapiravir	9	LYS551, ASP618 (2), LYS621 (2), SER795	VAL166, GLU167, PRO620		
	Fisetin	11	PHE442 (2), GLN444, ILE548, SER549, ARG553 (2)	LYS545, LYS551 (2), ARG553		
	Hyperoside	13	HIS439, SER549, ALA550, LYS551, GLN815 (2), ASP833, ARG836	ALA550, LYS551, ARG836		HIS439
**NSP12 RNA Polymerase (NiRAN)**	Remdesivir (Reference)	11	ARG349, THR394, PHE396, ASN459 (2)	THR319, PRO323, PHE396 (2), VAL675, PRO677		
	Balapiravir	11	PHE321, ARG349, ASN459, LEU460, ASN628	VAL315, PRO323, ARG349, ARG457, VAL675, PRO677		
	Fisetin	11	THR319, LEU460, THR462 (2), ASN628 (2), MET629	THR319, GLU350		
	Hyperoside	16	THR246, ARG249, LEU251, SER255, THR319 (2), ARG349 (2)	ARG249, PRO461		
